# Top of the Basilar Syndrome: A Case Report of a Healthy Young Woman and Literature Review

**DOI:** 10.7759/cureus.44663

**Published:** 2023-09-04

**Authors:** José G Rivera, José L Moreno, Irma L Maldonado, Edgar Bravo, José C Padilla

**Affiliations:** 1 Internal Medicine, General Hospital León, León, MEX; 2 Research, University Quetzalcoátl Irapuato, León, MEX; 3 Research, University of Guanajuato, León, MEX; 4 Critical Care Medicine, General Hospital León, León, MEX; 5 Radiodiagnosis, General Hospital León, León, MEX

**Keywords:** brain steam infarctions, top of the basilar syndrome, acute ischemic stroke, cerebral infarction, young adult, basilar artery, posterior circulation stroke, generalized tonic clonic seizures

## Abstract

Top of the basilar syndrome (TBS) is defined as the presence of multiple ischemic lesions on magnetic resonance image (MRI) including more than two territories supplied by branches of the distal portion of the basilar artery, causing symptoms such as dizziness, diplopia, ataxia, and acute cognitive decline that can lead to quadriplegia and death. Diagnosing TBS is challenging because it can mimic other conditions such as thalamic hemorrhages or vertebrobasilar ischemia, and requires advanced imaging. Although the prognosis for these patients is poor, rehabilitation is essential for their recovery. This case describes a healthy 28-year-old woman who presented with headache, vomiting, and tonic-clonic seizures sent to the hospital with a stroke diagnosis.

## Introduction

Basilar artery occlusion accounts for 1% of all strokes, with an incidence of four patients per 100,000 per year [1]. Lesions consistent with top of the basilar syndrome (TBS) have been defined as the presence of multiple ischemic lesions on diffusion-weighted imaging (DWI) involving more than two territories supplied by branches of the distal portion of the basilar artery (cerebellum, hypothalamus, medial diencephalon, and medial thalamus) and occlusion of the distal one-third of the basilar artery (BA) on both time-of-flight magnetic resonance angiography (MRA) and contrast-enhanced MRA [2].

Conventional risk factors typically include hypertension, diabetes, atrial fibrillation, smoking, and obesity. However, 30% of strokes remain of unknown origin, with cryptogenic stroke being particularly significant in individuals younger than 45 years [3].

TBS is due to a distal basilar occlusion clinically characterized by a decreased level of consciousness and somnolence due to injury to the bilateral thalami, ipsilateral complete facial palsy due to impairment of the seventh cranial nerve, vertical gaze, and convergence disturbances, resulting in pseudoparesis of the abducens nerve and peduncular hallucinosis [1,4].

The purpose of this article is to present a clinical case and literature review of a condition with a low incidence and limited reports, particularly in elderly patients and those without a significant medical history related to the development of the syndrome. However, given our patient's background, the study of risk factors for autoimmune or rheumatologic diseases and anatomic malformations required a meticulous, multidisciplinary diagnostic approach in the shortest possible time.

## Case presentation

**Figure 1:**
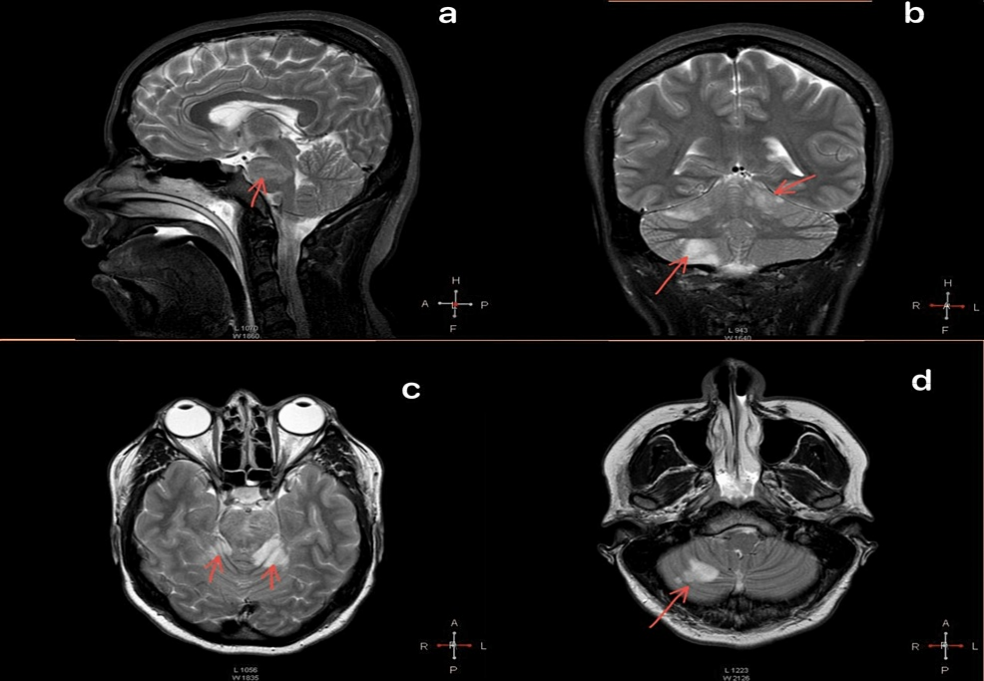
Sagittal, coronal, and axial magnetic resonance image a: Sagittal T2 magnetic resonance (MR) showing pons hyperintensity. b: Coronal T2 MR with diffuse hyperintensities in cerebellar hemispheres with more significant right extension. c: Axial T2 MR, diffuse hyperintensities in both cerebellar hemispheres and diffusely in the pons. d: Axial T2 MR showing diffuse hyperintensity in the right cerebellar hemisphere, compatible with an ischemic event of the posterior circulation.

**Figure 2:**
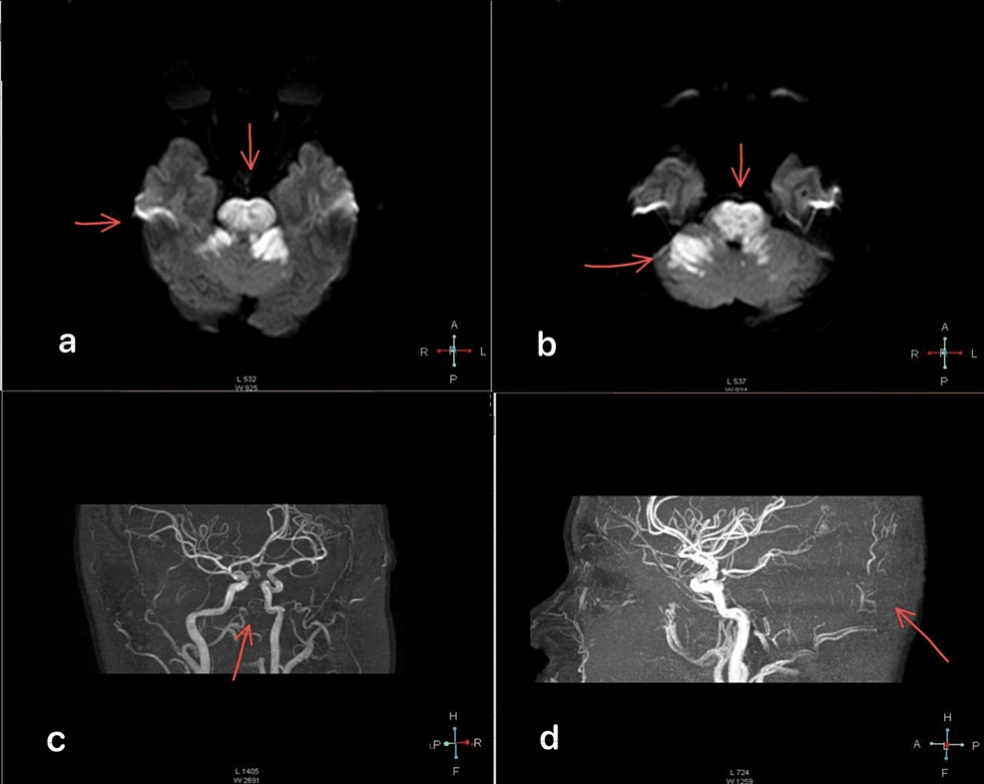
Magnetic resonance image (MRI) in DWI and magnetic resonance angiographic (a) DWI (diffusion-weighted imaging) MRI showing extensive restricted areas in the pons and cerebellar hemispheres. (b) DWI MRI in lower sections continuing to display these restricted areas. (c, d) Magnetic resonance angiographic (MRA) reveals the absence of vascular pathways in the posterior circulation, with no visualization of flow in the basilar artery or its branches.

A 28-year-old female patient with a history of hypertension diagnosed two months ago and poor adherence to treatment, no history of smoking, drug abuse, use of oral contraceptives, or other substances with no other relevant medical history, presented with oppressive occipital headache without radiation. Vomiting, generalized tonic-clonic seizures, and subsequent deterioration of consciousness followed the headache. The patient did not receive reperfusion therapy or thrombectomy within the therapeutic window because she arrived at our hospital three days after the onset of symptoms. She was diagnosed but not treated due to the high cost in a private clinic. The patient received neurocritical care in our hospital including orotracheal intubation and advanced airway management. A computed tomography (CT) scan of the head was performed and showed no structural abnormalities. The hospital performed magnetic resonance imaging (MRI), which showed evidence of stroke in the pons and cerebellum (Figure 1 and Figure 2). The patient was admitted to the intensive care unit with a diagnosis of "top of the basilar syndrome" of undetermined etiology due to the incomplete evaluation.

Consequently, an etiologic approach to the stroke was performed, which revealed the following initial findings: (a) transthoracic echocardiogram (TTE) showing hypertensive heart disease with a left ventricular ejection fraction of 52%, the TTE found no evidence of a patent foramen ovale (PFO) or other anatomical abnormalities that would indicate a compromise of venous or arterial flow; (b) carotid Doppler ultrasound showing atherosclerotic plaque in the carotid bulb without cerebral hemodynamic repercussions and normal vertebral arteries with unaltered velocity and waveform. After excluding major cardiac or circulatory pathologies, vasculitis or autoimmune etiology was suspected. The following laboratory tests were performed: Prothrombin time (PT 11.3 seconds), partial thromboplastin time (PTT 27.20 seconds), INR 1.03, protein C of coagulation (108%), protein S of coagulation (80%), anticardiolipin antibodies (IgM < 2 MPLU/mL and IgG 7.17 GPLU/mL), rheumatoid factor (less than 8.6 IU/mL), antinuclear antibodies (less than 0.5 INDEX), and anti-DNA (4.98 IU/mL). However, the specific etiology needed to be clarified, and a series of laboratory and imaging studies were performed at the hospital to identify to rule out the main etiologies. However, as the patient improved and was no longer under the care of our hospital, we were unable to continue the search for the etiology. Due to clinical improvement, the patient was transferred to the internal medicine ward. Sedation was gradually tapered, and extubation was performed. During the hospitalization, the patient experienced quadriplegia with recurrent muscle spasms in the extremities but retained hearing and vertical eye movements, while horizontal movements were impossible. The patient was eventually sent home.

## Discussion

Background

TBS also known as rostral brainstem infarction, was first described by John Abercrombie in 1828 when he reported the case of an 18-year-old man whose autopsy revealed "ossification" of the BA [5]. Later, in 1980, Caplan conducted a review that focused on the neurological abnormalities of patients with infarction in the area he termed the "top of the basilar," although he still did not know the exact location or mechanism by which infarction occurred in this area [6].

Epidemiology

Stroke secondary to BA thrombosis represents 20% of all posterior circulation cerebrovascular accidents and 1-4% of all ischemic strokes. Ahn et al. reported an incidence of 8.5% of TBS in a population of who experienced acute ischemic stroke in the posterior circulation during the study [2]. Borelli Del Guerra et al. conducted a study on mortality in patients who did not receive reperfusion therapy, resulting in an in-hospital mortality rate of 45.4% and a cumulative mortality rate of 63.6% at the last follow-up [7].

Risk factors

Rostral BA occlusion has been previously described in cerebrovascular accidents caused by large-vessel disease and atherosclerosis, cardiovascular embolism, cervical and intracranial vertebral artery dissection, or unknown causes [8]. Often, BA occlusion shares the same cardiovascular risk factors as stroke. However, occlusion can be caused by different mechanisms, the two most common being cardioembolic (36%) and large vessel atherosclerosis (35%), with other mechanisms, such as aortic dissection and vasculopathy, accounting for a smaller percentage of cases [1].

Following the mentioned risk factors, we performed a literature review to look for similar cases, identifying the principal risk factors and the age of the patients, as summarized in Table 1.

**Table 1:** Reported cases of top of the basilar syndrome

Country	Year	Sex	Age	Abstract
United States	2020	Male	38 years	The patient presented to the emergency department with generalized stiffness and altered consciousness, which was attributed to hyperreflexia caused by a brainstem stroke secondary to thromboembolism in the basilar artery due to dissection of the right vertebral artery. The patient received thrombectomy and started on therapeutic anticoagulation, Three months later the patient had continued to improve. His cognition and language were normal [8].
United States	2020	Female	64 years	A patient with a history of cardiac arrest, currently on a pacemaker, presented to the emergency department with the inability to speak and altered consciousness secondary to a thrombotic occlusion at the top of the basilar artery resulting in infarction of the medial thalamus and cerebellum, she was under mechanical ventilation and treated with clopidogrel and aspirin, underwent tracheostomy and gastrostomy on day 9, adding sodium valproate to her antiepileptic regimen resulted in suppression of epileptiform activity. She became more alert and responsive at that point, though she remained nonverbal and only followed simple commands. On hospital day 33, she was discharged to a long-term acute care facility [9].
United States	2021	Female	19 years	A patient admitted to the emergency department after a motor vehicle accident where she developed a subdural hematoma with mass effect and persistent intracranial hypertension, emergent decompressive hemicraniectomy and ventriculostomy placement were undertaken, she developed sustained refractory intracranial hypertension starting two days postoperatively resulting in a top of the basilar syndrome involving the thalamus, midbrain, and posterior cerebral artery territory, The patient required tracheostomy and gastrostomy placement, as well as treatment of central panhypopituitarism. She was eventually discharged to a care facility in a condition of persistent unresponsive wakefulness [10].
United States	2020	Female	36 years	A patient treated with oral contraceptives, morbidly obese, and taking weight loss supplements presented with dizziness, vomiting, headache, and neck pain accompanied by paresthesia of the left lip. Symptoms progressed to left eye ptosis, right medial gaze palsy, and right upper extremity dysmetria. MRI showed acute infarcts in the right midbrain, right pons, and left cerebellar hemisphere, consistent with basilar artery thrombosis. The patient was transferred to a tertiary care center for emergent endovascular thrombectomy. Following successful thrombolysis in cerebral infarction (TICI) grade 3 thrombectomy, the patient experienced a significant improvement of her symptoms, with only mild right facial droop and right internuclear ophthalmoplegia noted on physical exam [11].
Italy	2023	Female	76 years	The patient was admitted to the emergency department after experiencing dizziness followed by loss of consciousness. An MRI was performed, which revealed an infarct in the paramedian thalamus and mesencephalon caused by occlusion of the basilar artery. The patient underwent systemic thrombolysis and mechanical thrombectomy, with complete reperfusion of the basilar artery. After the procedure, the patient was alert and oriented, and her examination demonstrated apraxia of eyelid opening (ALO) and vertical-gaze palsy [12].

Etiology

The most common causes of BA occlusion are atherosclerotic occlusion (26-36%) resulting from local thrombosis due to severe stenosis and embolic occlusion (30-35%) of cardiac or large vessel origin. Other less common causes include vertebral artery dissection (6-8%) and undetermined causes (22-35%) [10]. Occlusions involving the distal BA are more often cardioembolic in nature [4]. Some of the etiologies described for TBS or rostral BA occlusion in the context of stroke are secondary to large or small vessel arterial disease, cardiovascular embolism, and even unknown causes. In young patients, common etiologies are related to a hypercoagulable state, such as hyperhomocysteinemia, antiphospholipid syndrome, non-neoplastic blood viscosity disorders, iron deficiency, and hepatitis C-associated cryoglobulinemia. Factor deficiencies are only rarely performed because their association with arterial thrombosis remains uncertain [13].

Pathophysiological mechanism

The mechanism underlying the characteristic clinical presentation involves a decrease in consciousness due to ischemia of the reticular activating system in the thalamus. Quadriplegia or hemiparesis is secondary to the involvement of the corticospinal tracts, and cranial nerve dysfunction is due to the involvement of the brainstem, the mesencephalic nucleus, or the direct involvement of the cranial nerves [1].

A study reported by de Vicino et al. investigated various mechanisms involved in stroke. The results showed that vasculitis was the most common mechanism (11.7%), followed by non-inflammatory vasculopathy (10.8%), hematologic abnormalities (6.3%), cancer (22.5%), rare cardiac mechanisms (10.8%), hemodynamic abnormalities such as hypotension (3.6%), issues related to diagnosis and therapeutic interventions (25.6%), and genetic diseases (2.2%) as some of the less common mechanisms involved in the development of stroke [14].

Signs and symptoms of TBS

This syndrome is characterized by sudden loss of consciousness due to injury to the bilateral thalami. Vision loss including cortical blindness is common. In addition, consciousness is preserved but has visual deficits that are often associated with pupillary and oculomotor dysfunction, including vertical gaze and convergence disorders and spasms resulting in pseudo-paresis of the abducens nerve [1].

Some of the most frequently registered symptoms also include blurry vision, unilateral limb weakness, dizziness, and headache, other symptoms less frequent are limb sensory deﬁcit, dysarthria, nausea, lethargy, cranial nerve V symptoms, and diplopia, loss of consciousness, dysphagia, and hearing loss [8].

Furthermore, a study by Cheng et al. documented that 0.43% of patients with TBS presented with a first manifestation of seizure episodes, and of these, 25.7% experienced a posterior circulation stroke [15].

Diagnostic approach

The etiology of stroke was defined according to the Trial of Org 10.172 in Acute Stroke Treatment (TOAST) classification, it was determined as a stroke of undetermined etiology due to the negative results obtained from the examinations and tests performed [7].

The initial evaluation of patients suspected of TBS includes non-contrast CT and CT angiography (CTA). On non-contrast CT, hyperdensity of the BA is often an indicator of BA occlusion with a sensitivity of 81% and specificity of 91%. In addition, CTA is recommended as an initial diagnostic modality because it is usually quick and easy to perform, has high sensitivity, and helps discriminate between TBS due to embolism and atherosclerosis of the large arteries. The analysis of CTA source imaging signiﬁcantly increases the performance of CT for detecting ischemic changes from 21-46% to 27-65% and improves the prognostic value of posterior circulation acute stroke prognosis early computed tomography scores (pc-ASPECTs). DWI on MRI is considered the gold standard for evaluating posterior fossa ischemia. As a complementary tool, the addition of Fluid-Attenuated Inversion Recovery (FLAIR) MRI has been suggested to determine whether the tissue is ischemic or infarcted. As for the initial CT imaging, adding perfusion-weighted imaging (PWI) increases MRI sensitivity [1,4].

Treatment

The treatment is reperfusion. In the study by Yan et al. patients with TBS had a good outcome with intravenous thrombolysis (IVT) and those who received endovascular therapy (EVT) had a higher rate of recanalization (98.3% vs. 76.6%, p < 0.001). Depending on the etiology, some patients have a better outcome. In patients with other types of BA occlusion and reperfusion therapy, those who received EVT achieved a significantly higher rate of recanalization. For example, patients who received EVT for BA occlusion secondary to embolism (cardiac or large vessel) had a better result than those with intracranial atherosclerotic disease or atherosclerotic thrombosis [16,17].

Outcomes

Although there have been recent advances in the treatment of acute stroke, up to 68% of patients with BA occlusion die or are significantly disabled [18]. Extensive infarction may be an indicator of poor prognosis regardless of successful revascularization, in contrast to patients with limited infarct territory who may have a higher probability of benefiting from reperfusion therapy [1].

Sennfalt et al. conducted a longitudinal observational study and reported a 30-day survival rate of 88.9% with a five-year cumulative survival rate of 49.4%. Puetz et al. reported poor functional outcomes in 70% of their patients with BA occlusion. At one month, 25% had a favorable outcome, 31% survived with an unfavorable outcome, and 44% died [19,20].

In the study by Borelli Del Guerra et al., favorable outcomes were observed in 35.7% of patients, 47% of whom received reperfusion therapy and 18.1% of whom did not [7].

## Conclusions

Diagnosis of TBS is challenging due to the similarity of symptoms to other diseases such as bilateral ischemic strokes, brainstem hemorrhage, seizures, and post-ictal state. It is essential to use advanced imaging techniques and a diagnostic approach in young patients with cerebrovascular disease to identify embolic or cardioembolic risk factors and other pathologies. Despite the poor prognosis, rehabilitation is crucial for recovery. Treatment includes reperfusion therapy and, outside the time window, supportive measures to prevent further injury. Rapid recognition of this rare condition is essential for prompt treatment and damage reduction.
